# Case Report: IVC-agenesis and FVL mutation; successful DVT/PE treatment with direct oral anticoagulation (factor Xa inhibitor)

**DOI:** 10.3389/fcvm.2025.1458064

**Published:** 2025-03-26

**Authors:** Abid Siddiqui, Tara Hatfield, Quynh Nguyen, Waris Waris

**Affiliations:** ^1^ West Virginia School of Osteopathic Medicine, Lewisburg, WV, United States; ^2^ Department of Hematology-Oncology, Logan Regional Medical Center, Logan, WV, United States; ^3^ Department of Internal Medicine, Logan Regional Medical Center, Logan, WV, United States

**Keywords:** inferior vena cava agenesis, factor V Leiden, deep vein thrombosis, pulmonary embolism, direct oral anticoagulant

## Abstract

Inferior vena cava (IVC) agenesis is a rare congenital anomaly that has been implicated in up to 5% of unprovoked deep vein thrombosis (DVT) cases in young men under 30 years old. We present the case of a 28-year-old obese Caucasian male who arrived at our hospital with significant pain and swelling in his right lower extremity. The patient had no prior medical history or family history of DVT or cardiovascular conditions. A venous Doppler ultrasound revealed an extensive right lower extremity DVT. Further imaging with a computed tomography (CT) pulmonary embolism (PE) protocol scan of the chest and abdomen identified IVC agenesis along with pulmonary emboli in the left central pulmonary arteries. A hypercoagulability workup was positive for a heterozygous Factor V Leiden (FVL) mutation, an additional thrombophilic risk factor. The patient was initially managed with an intravenous heparin drip and was later transitioned to long-term direct Factor Xa inhibitor therapy. To our knowledge, this is the first reported case of extensive venous thromboembolism (VTE) due to concurrent IVC agenesis and FVL mutation successfully treated with direct Factor Xa inhibition. This case highlights the complexity of managing patients with multiple thrombogenic risk factors and raises a discussion on the rationale for lifelong anticoagulation in such individuals.

## Introduction

Inferior vena cava (IVC) agenesis is a rare congenital anomaly with its prevalence ranging between 0.0005% and 1% (1). Though rare, it is still a cause for concern since literature has shown as much as 5% of young men under the age of 30 who suffer from unprovoked DVTs are affected by IVC agenesis (2). This condition results from embryonal dysgenesis during the prenatal stage (3). The absence of an IVC leads to the development of extensive collateral veins to maintain adequate blood flow to the heart. This compensatory mechanism, however, increases the risk of venous thromboembolisms (VTE), including deep vein thrombosis (DVT) and pulmonary embolisms (PE). VTEs are critical conditions that can result in severe complications, including stroke, PE, and sudden death.

Inherited thrombophilias such as the heterozygous factor V Leiden (FVL), further amplify VTE risk by production of a factor V protein that is resistant to inhibition by Protein C. Research suggests that individuals with a heterozygous FVL mutation have a 6–8-fold increased risk of developing VTE, while those with a homozygous mutation face an 80-fold increase (4). The coexistence of IVC agenesis alongside inherited thrombophilia complicates the clinical management of affected VTE patients, and underscores the importance of effective treatment strategies to mitigate the risk of life- threatening complications.

## Case description

A 28 year old male arrived at our hospital with a one day history of right lower extremity swelling and pain. Patient stated it was painful to move his right calf muscle, and that he had a general feeling of tightness around it. He denied any inciting incidents that could have caused the symptoms and stated that walking on the leg worsened it. He denied chest pain, shortness of breath, and any personal or family medical history of cardiovascular related diseases, including DVTs. He also stated he was not currently taking any medications, though his past medical history included depression, anxiety, and seizures. His past surgical history included a right shoulder surgery and vagal nerve stimulator implant. He denied any history of tobacco, alcohol, illicit drug use.

Upon physical examination, we found that our patient had swelling of his right lower extremity with a purple discoloration of his overlying skin of the affected region. The swelling extended from his right ankle up to the lower aspect of his right knee. He exhibited no deficits to motor strength and sensation across his extremities. Auscultation of his heart and lungs was unremarkable. Strong dorsalis pedis pulses bilaterally and brisk capillary refill times were appreciated. His abdomen was soft, and non-tender with normal bowel sounds. Overall neuropsychiatric status was unremarkable with normal affect, judgment/insight.

His vital signs were relatively stable; pulse oximetry measured 99% O2 saturation, with a blood pressure of 127 systolic over 66 diastolic. His pulse was 82 beats per minute, and his respiratory rate was at 17 per minute. His temperature was 98.2 degrees Fahrenheit.

**Figure 1:**
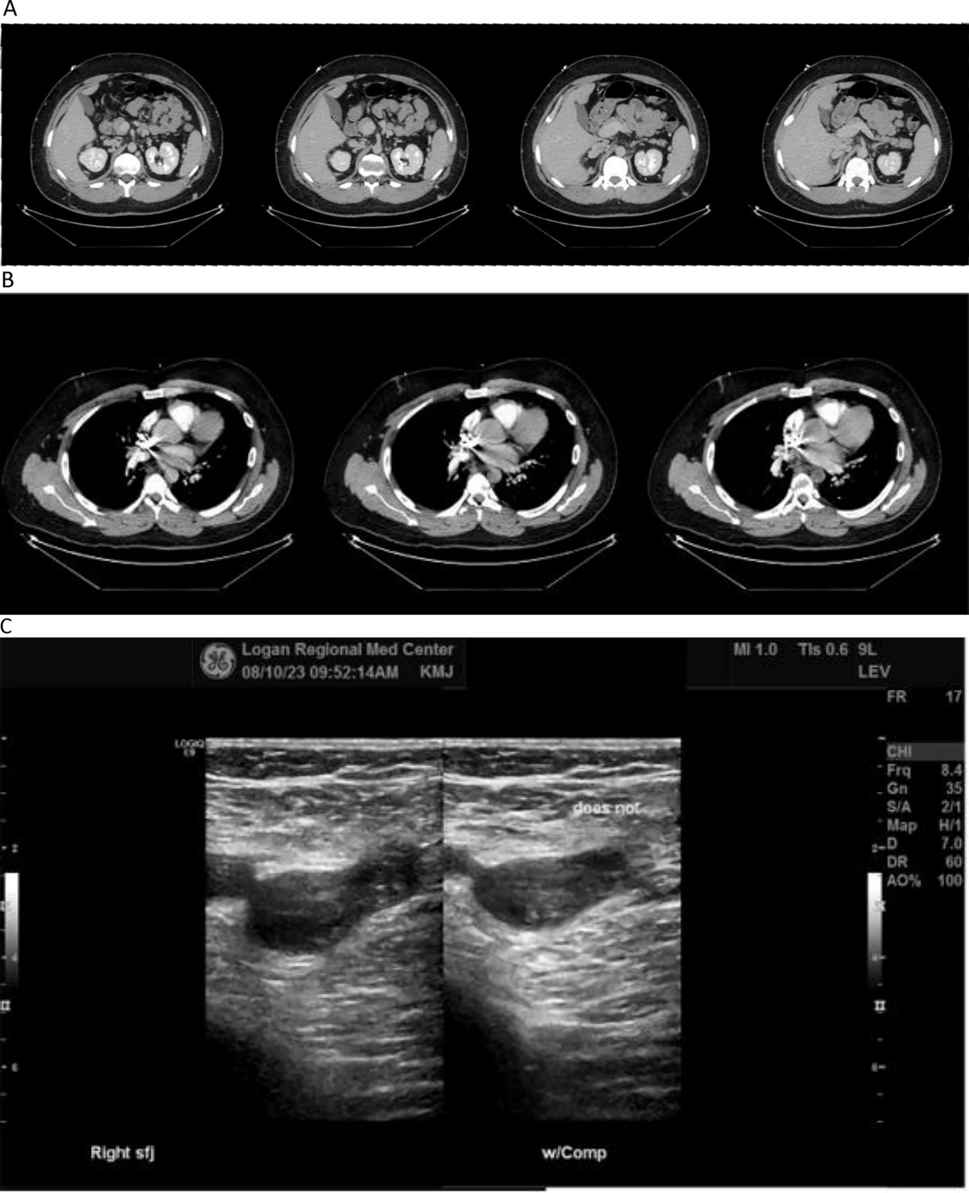
**(A–C)** diagnostic imaging. **(A)** CT of abdomen with contrast. **(B)** CT of chest with contrast. **(C)** Venous doppler ultrasound of right leg. Contrast-enhanced CT scans of patient's abdomen **(A)** and chest **(B)** showing evidence of pulmonary emboli in left central pulmonary arteries, and agenesis of the inferior vena cava with extensive collateralization. Venous Doppler ultrasound of the right lower extremity **(C)** showing thrombus present from the common femoral vein distally into the popliteal vein extending into the greater saphenous vein.

Initial diagnostic workup included a venous doppler ultrasound of the right lower extremity, which yielded findings of thrombus extending from the common femoral vein distally into the popliteal and greater saphenous veins (Figure 1C).

Comprehensive metabolic panel demonstrated normal kidney function and electrolyte levels, with slightly elevated ALT reading of 67 units/L (Tables 1–3). Blood analysis demonstrated elevated mean platelet volume and monocyte percentage, alongside low lymphocyte percentage of his total white blood cell composition (Tables 1–3).

**Table 1:** Complete blood count (CBC) with differential.

Test	Result	Reference ranges
WBC	9.1 thousand/mm^3^	4.8–10.8 thousand/mm^3^
RBC	4.61 million/mm^3^	4.2–5.4 million/mm^3^
Hemoglobin	14.1 g/dl	14–18 g/dl
HCT	42.4%	42.0%–52.0%
MCV	92.0 fL	78–98 fL
MCH	30.6 pg	27–31 pg
RDW	12.5%	10.7%–14.0%
Platelet count	198 k/mm^3^	130–400 k/mm^3^
MPV	9.4 fL	6.6–9.3 fL
Gran %	72.4%	42.2%–75.2%
Lymphocyte %	15.6%	20.5%–51.1%
Monocyte %	10.6%	1.7%–9.3%
Eosinophil %	0.8%	0.0%–10.0%
Basophil %	0.3%	0.0%–0.8%

**Table 2:** Comprehensive metabolic profile.

Test	Result	Reference ranges
Glucose	92 mg/dl	70–110 mg/dl
BUN	13 mg/dl	7–18 mg/dl
Creatinine	0.8 mg/dl	0.6–1.3 mg/dl
eGFR	123.63	

**Table 3:** Electrolyte panel, and liver enzyme measurements.

Test	Result	Reference ranges
Sodium	138 mmol/L	135–145 mmol/L
Potassium	4.2 mmol/L	3.5–5.1 mmol/L
Chloride	104 mmol/L	98–107 mmol/L
CO2	26.6 mmol/L	21–32 mmol/L
Calcium	9.1 mg/dl	8.5–10.1 mg/dl
Total protein	7.6 gm/dl	6.4–8.2 gm/dl
Albumin	4.0 gm/dl	3.4–5.0 gm/dl
Bilirubin, total	0.6 mg/dl	0.0–1.0 mg/dl
SGOT/AST	24 Units/L	15–37 Units/L
SGPT/ALT	67 Units/L	30–65 Units/L
Alkaline phosphatase	68 Units/L	50–136 Units/L

Hematologic workup yielded a heterozygous FVL mutation (c. 1601G>A, p.Arg534Gln). Patient tested negative for IgA, IgG, and IgM antibodies against Beta-2 glycoproteins, and cardiolipin. He also tested negative for abnormal levels of homocysteine in his serum.

Patient consented to further investigation involving contrast-enhanced CT scans of his chest which yielded filling defects within the central pulmonary arteries on the left consistent with pulmonary emboli (Figure 1B). Abdominal CT scan showed agenesis of the inferior vena cava with extensive collateralization (Figure 1A), and enlargement of the right common iliac and femoral veins consistent with DVT found on ultrasound (Figure 1C).

After DVT was confirmed via doppler ultrasound, patient was immediately started on a standard Heparin weight-based IV drip. Once further work-up and labs were completed, a decision was made to transition patient from IV Heparin to oral anti-coagulation utilizing Apixaban 5 mg twice-daily upon discharge.

Since his discharge with the new diagnosis, our patient has remained symptom-free for 13 months, with no recurrence or worsening of his DVT or PE. The patient's right lower extremity swelling has completely resolved, and he has not reported any new episodes of shortness of breath, chest pain, or other complications associated with his pulmonary embolism. He has successfully resumed his daily activities, including the physical demands of his nursing studies, with significantly reduced pain and swelling. His ability to walk, stand for prolonged periods, and participate in clinical rotations has markedly improved, allowing him to progress in his training without significant limitations.

Given the congenital nature of his IVC agenesis and the associated risk of recurrent thromboembolic events, our multidisciplinary team—including specialists from interventional radiology, vascular surgery, and hematology—agreed that long-term anticoagulation therapy remains the most appropriate management strategy. Due to the complexity of his vascular anatomy, thrombectomy was deemed unsuitable, and no viable surgical interventions are currently available. Therefore, the decision was made to continue anticoagulation therapy indefinitely with Apixaban 5 mg twice daily to minimize the risk of further thrombotic events.

We continue to monitor the patient closely through outpatient follow-ups every three months, assessing for potential complications such as recurrent DVTs, post-thrombotic syndrome, or anticoagulation-related adverse effects. As newer mechanical interventions become available, we will reassess treatment options in collaboration with our multidisciplinary team to determine if a more definitive approach to managing his IVC agenesis emerges.

## Discussion

In managing a patient with a heterozygous FVL mutation without prior VTE history, education and routine care are typically sufficient. Management of an acute VTE in the setting of an inherited thrombophilia involves anticoagulation for at least three to six months. Indefinite anticoagulation is a decision that is subject to the physician's clinical judgment, based on various risk factors on a case by case basis including multiple thrombophilic mutations, strong family history of VTE, anatomically extensive DVT, and life- threatening PE. In our patient's case, he has both a heterozygous Factor V Leiden deficiency and IVC agenesis. He also had an extensive DVT on his right side extending from his great saphenous vein to femoral vein, and PE. Current guidelines are lacking for this complex situation.

Current literature demonstrates that two groups encountered similar challenges in managing IVC agenesis in their patients. In 2014, Lamparello et al. managed a patient with DVT, IVC agenesis, and FVL mutation with indefinite vitamin K antagonist therapy (3). In 2019, Estevez Cruz et al. described treating a patient with DVT and IVC agenesis using a direct Factor Xa inhibitor indefinitely, reporting no new DVT events over four years of follow-up (5). Though the two groups (Estevez Cruz, and Lamparello) ultimately discharged their patients with different treatment regimens, both reported no established guidelines in medical literature of what anticoagulant to utilize.

The decision to discharge our patient on direct Factor Xa (Apixaban) was largely due to the difficulty in managing Vitamin K antagonist (VKA) treatment. Experts recommend patients spend at least 65% of their VKA treatment in the appropriate therapeutic range (calculated as TTR), but a review in 2016 by Schein et al., found patients on average spent ∼54%–65% in the TTR appropriate range (6). Maintaining the target therapeutic range for VKA treatment, like Warfarin, is challenging due to factors like missed appointments, doses, and drug interactions. Deviations from the ideal INR range (2, 3) can lead to thrombotic events or severe bleeding, including gastrointestinal and intracranial hemorrhages.

Our decision to select Apixaban over other direct oral anticoagulant (DOAC) drugs, and VKAs was attributed to its low-risk comprehensive safety profile. In a review by Ballestri et al. published in 2023, it was cited that one study found DOAC drugs reduced the risk of intracranial hemorrhage by over 40% compared to VKAs (specifically, Apixaban reduced the risk by 57%, while Rivaroxaban did by 41%) (7, 8). The review also cites Apixaban as having the best safety profile for GI bleeds compared to VKAs and other DOACs (7).

A recent study by Bravo-Perez et al. published in 2024 analyzed 122 patients with IVC agenesis and venous thrombosis, identifying 18 patients (16%) with an FVL mutation, underscoring a potential link between congenital vascular anomalies and inherited thrombophilia (9). Most patients (89%) were initially treated with vitamin K antagonists (VKAs), while a smaller subset received direct oral anticoagulants (DOACs) as frontline therapy (7 patients) or were switched from VKAs to DOACs after initial treatment (16 patients). Another 7 patients remained on low-molecular-weight heparin (LMWH) without transitioning to oral therapy. The study reported a 40% recurrence rate of venous thrombosis, with recurrence significantly higher (51%) in those who discontinued anticoagulation compared to those on indefinite therapy (35%). Recurrence was defined as radiologically confirmed new thrombotic events at a different site from the initial clot, accompanied by clinical symptoms. Among patients with both IVC agenesis and FVL mutation, inherited thrombophilia was significantly associated with a higher risk of recurrent thrombosis (aOR 3.19, 95% CI 1.09–9.32, *p* = 0.034). Overall, although IVC-agenesis serves as a congenital anomaly that may appear as a primary driver of thrombosis, we still believe based on the statistics published in this study that it further stresses the importance of evaluating genetic and image-based thrombophilia testing in all young patients with unprovoked DVTs. The study commented on how often simple and easy steps undertaking abdominal imaging can help discover previously unknown congenital anomalies like IVC-agenesis that can make a life-changing difference in a first time unprovoked DVT patient. We also believe that this landmark study evaluating an otherwise difficult to find dataset of 122 patients supports the importance of having IVC-agenesis patients remain on life-long anticoagulation to help reduce the risk of recurrent thrombosis.

Indefinite anticoagulation remains the most effective strategy for reducing recurrent thrombotic events in patients with IVC agenesis, particularly those with additional risk factors such as FVL mutation. Given the high recurrence risk in this population, lifelong anticoagulation with a well-tolerated DOAC like Apixaban offers the best balance between efficacy and safety. However, as endovascular and surgical interventions evolve, future studies are needed to explore alternative treatment options that may provide a more definitive solution for patients with congenital vascular anomalies like IVC agenesis.

## Conclusion

The coexistence of IVC-agenesis and heterozygous FVL mutation in a young patient can lead to unprovoked DVT, and PE development. This case underscores the rarity and complexity of treating patients with multiple predisposing factors for VTE. The successful use of direct oral Factor Xa oral anticoagulation (Apixaban) in this scenario highlights its potential as a viable long-term treatment option, opening new discussions for managing similar complex cases in the future.

## Data Availability

The original contributions presented in the study are included in the article/Supplementary Material, further inquiries can be directed to the corresponding author.
